# 2,3,7,8-Tetrachlorodibenzo-*p*-dioxin (TCDD) dysregulates hepatic one carbon metabolism during the progression of steatosis to steatohepatitis with fibrosis in mice

**DOI:** 10.1038/s41598-020-71795-0

**Published:** 2020-09-09

**Authors:** Russell R. Fling, Claire M. Doskey, Kelly A. Fader, Rance Nault, Tim R. Zacharewski

**Affiliations:** 1 Microbiology and Molecular Genetics, Michigan State University, East Lansing, MI 48824 USA; 2 Biochemistry and Molecular Biology, Michigan State University, East Lansing, MI 48824 USA; 3 Institute for Integrative Toxicology, Michigan State University, East Lansing, MI 48824 USA

**Keywords:** Metabolomics, Gene expression, Gene regulation, Mechanisms of disease, Transcriptomics

## Abstract

2,3,7,8-Tetrachlorodibenzo-*p*-dioxin (TCDD), a persistent environmental contaminant, induces steatosis that can progress to steatohepatitis with fibrosis, pathologies that parallel stages in the development of non-alcoholic fatty liver disease (NAFLD). Coincidently, one carbon metabolism (OCM) gene expression and metabolites are often altered during NAFLD progression. In this study, the time- and dose-dependent effects of TCDD were examined on hepatic OCM in mice. Despite AhR ChIP-seq enrichment at 2 h, OCM gene expression was not changed within 72 h following a bolus dose of TCDD. Dose-dependent repression of methionine adenosyltransferase 1A (*Mat1a*), adenosylhomocysteinase (*Achy*) and betaine-homocysteine S-methyltransferase (*Bhmt*) mRNA and protein levels following repeated treatments were greater at 28 days compared to 8 days. Accordingly, levels of methionine, betaine, and homocysteic acid were dose-dependently increased, while S-adenosylmethionine, S-adenosylhomocysteine, and cystathionine exhibited non-monotonic dose-dependent responses consistent with regulation by OCM intermediates and repression of glycine N-methyltransferase (*Gnmt*). However, the dose-dependent effects on SAM-dependent metabolism of polyamines and creatine could not be directly attributed to alterations in SAM levels. Collectively, these results demonstrate persistent AhR activation disrupts hepatic OCM metabolism at the transcript, protein and metabolite levels within context of TCDD-elicited progression of steatosis to steatohepatitis with fibrosis.

## Introduction

One carbon metabolism (OCM) comprises the interlinking methionine and folate cycles to provide one carbon units for biosynthetic reactions^1^. This includes the biosynthesis of S-adenosylmethionine (SAM), the primary cellular methyl donor for methyltransferase reactions, and the second most utilized enzymatic cofactor after ATP^2^. SAM is essential for the biosynthesis of several products required for maintaining and regulating cell structure and function including creatine for ATP regeneration, phospholipids such as phosphatidylcholine for membrane integrity and lipid transport, and epigenetic gene regulation via the methylation of histones, DNA, and RNA^3,4^. In addition, decarboxylated SAM serves as the source of aminopropyl groups for polyamine biosynthesis which is important for cell growth, survival and proliferation^5^.

Alterations in the levels of SAM, as well as related OCM metabolites, can have profound effects on cell growth, differentiation, response to injury, and tissue regeneration^1^. For example, SAM reduces homocysteine re-methylation by allosterically inhibiting betaine homocysteine methyltransferase (BHMT) in the methionine cycle and methylenetetrahydrofolate reductase (MTHFR) in the folate cycle while activating S-adenosylmethionine synthase isoform type-1 (MAT1A) and cystathionine β-synthase (CBS) in the transsulfuration pathway^6^. SAM also activates glycine N-methyltransferase (GNMT), a highly expressed hepatic enzyme that converts excess SAM levels to sarcosine to maintain methionine homeostasis^6^. Furthermore, methyltransferases are inhibited by the de-methylated product of SAM, S-adenosylhomocysteine (SAH). This includes GNMT which is regulated post-transcriptionally by phosphorylation and allosterically by 5-methyltetrahydrofolate^7^. Inhibition or reduction of OCM-related gene expression in humans or rodent models, as well as prolonged treatment of rodents with high fat diets, alcohol, or carbon tetrachloride, alter OCM metabolite levels during the development of chronic liver diseases comprising non-alcoholic fatty liver disease (NAFLD), cirrhosis, and hepatocellular carcinoma^8,9^. Interestingly, the severity of histopathological lesions in these disease models is alleviated following administration of SAM, betaine or creatine^10–12^. BHMT^13^, GNMT^14^, and MAT1A^15^ knockout models exhibit NAFLD phenotypes with steatosis often progressing towards fibrosis and hepatocellular carcinoma. Phosphatidylethanolamine N-methyltransferase (PEMT) knockout mice have altered phosphatidylcholine levels and steatosis as well as compromised very low density lipoprotein (VLDL) assembly and impaired secretion^16,17^. Phosphatidylcholine biosynthesis via PEMT is critical for hepatic lipid homeostasis, triglyceride packaging, and VLDL particle assembly prior to export.

NAFLD, defined as a spectrum of hepatic disorders, begins as simple steatosis that may progress to steatohepatitis with cirrhosis. Underlying NAFLD increases the risk for more complex metabolic diseases including metabolic syndrome, diabetes, cardiovascular disease, and hepatocellular carcinoma. NAFLD is estimated to affect ~ 35% of the U.S. population and has emerged as a leading cause for liver transplant^18–21^. In addition to genetics, age, lifestyle, diet and circadian dysregulation, accumulating evidence suggests environmental contaminants may play an underappreciated role in NAFLD development and progression. For instance, 2,3,7,8-tetrachlorodibenzo-*p*-dioxin (TCDD) and related compounds dose-dependently induce the progression of steatosis to steatohepatitis with fibrosis in mice^22–24^. Exposure to TCDD and related compounds is also associated with dyslipidemia and altered glucose homeostasis and liver function in human epidemiological studies^25–28^. These effects are mediated by the aryl hydrocarbon receptor (AhR), a ligand activated basic helix-loop-helix/Per-Arnt-Sim transcription factor. Interestingly, the severity of NAFLD associated histopathologies is absent in AhR-null (*Ahr* ^-/-^) models and reduced in heterozygous (*Ahr*^+/−^) models, as well as in mice expressing the weaker ligand binding *Ahr*^*d*^ allele^29–32^.

Previous studies have shown TCDD alters the hepatic expression of genes associated with dyslipidemia, inflammation, and fibrosis as well as the regulation of the circadian clock^22,23,33^. However, there are a paucity of reports investigating the effects of TCDD on OCM. In this study, we tested the hypothesis that TCDD would elicit dose-dependent changes in OCM gene expression, protein levels, and metabolite levels consistent with the progression of NAFLD pathologies in mice. Our results show TCDD dose-dependently altered OCM metabolism and the SAM/SAH ratio, but effects on polyamine and creatine associated gene expression and metabolites levels could not be directly attributed to impacts on cellular methylation potential.

## Materials and methods

### Animal treatment

Postnatal day 25 (PND25) male C57BL/6 mice weighing within 10% of each other were obtained from Charles River Laboratories (Kingston, NY) and housed and treated as previously described^22^. Briefly, mice were housed in Innovive Innocages (San Diego, CA) containing ALPHA-dri bedding (Shepherd Specialty Papers, Chicago, IL) in a 23 °C environment with 30–40% humidity and a 12 h/12 h light/dark cycle. Aquavive water (Innovive) and Harlan Teklad 22/5 Rodent Diet 8,940 (Madison, WI) were provided ad libitum. The rodent diet is a fixed formula complete diet with an energy density of 3.0 kcal/g and a nutrient ingredient composition including 22% protein, 5.5% fat, and 40.6% carbohydrate. Mice (PND29) were orally gavaged at the beginning of the light cycle with 0.1 ml sesame oil vehicle (Sigma-Aldrich, St. Louis, MO) or 0.01, 0.03, 0.1, 0.3, 1, 3, 10, and 30 μg/kg body weight TCDD (AccuStandard, New Haven, CT) every 4 days for either 2 (n = 5) or 7 exposures (n = 8). The first gavage was administered on day 0 of the study, while the final gavage was on day 4 and day 24 for the 8- and 28-day studies, respectively. On day 8 or day 28, vehicle- and TCDD-treated mice (fasted for 6 h with access to water) were weighed and euthanized. For time course work, male mice were orally gavage with a single bolus dose of 30 µg/kg TCDD and samples collected at 0, 2, 4, 8, 12, 24 and 72 h (n = 5). Upon collection, liver samples were immediately flash frozen in liquid nitrogen. Collected tissues were stored at -80 °C until analysis. All animal handling procedures were performed with the approval of the Michigan State University (MSU) Institutional Animal Care and Use Committee, in accordance with ethical guidelines and regulations.

### Metabolite standards

S-adenosylmethionine-^13^C_5_ (#A291533)**,** N1-acetylspermidine (#A187845**)**, putrescine-d8 (#D416027), spermidine-d6 (#S680407), spermine-d20 (#S680512) were purchased from Toronto Research Chemicals (Toronto, Ontario, Canada). S-Adenosylhomocysteine-d4 (#13603), spermidine (#14918) and spermine (#18041) were purchased from Cayman Chemical (Ann Arbor, MI). Putrescine (D13208), homocysteic acid (#44925), *N*,*N*-dimethylglycine (#05022), amino acid standards solution (A9906), and the cell free amino acid mixture-^13^C,^15^ N (#767964) were purchased from Sigma-Aldrich. Standard calibration curves between 0.01 and 10 µM were constructed using serially diluted unlabeled standards with internal standards at 2 µM.

### Metabolite extraction and sample processing

Metabolites were extracted from frozen liver samples using methods optimized for the specific metabolites of interest. SAM, SAH and one carbon metabolites (betaine, cystathionine, homocysteic acid, L-methionine, and N,N-dimethylglycine) were extracted from frozen liver using perchloric acid (PCA). Briefly, ~ 25 mg liver tissue was added to ice cold 0.4 M PCA containing internal standards (SAM-^13^C_5_, SAH-d4, and cell free amino acid mixture-^13^C,^15^N) and homogenized for 15 s using a Polytron PT2100 homogenizer (Kinematica AG, Luzern, CH). The mixture was centrifuged for 10 min at 13,000×*g*, after which the supernatant was removed, and the protein pellet was saved for protein quantification. Supersaturated potassium bicarbonate was added to the supernatant and centrifuged at 13,000×*g* at 4 °C. Half of the supernatant was removed (335 µl) for SAM & SAH quantification and diluted with perfluoroheptanoic acid to a final solution of 10 mM perfluoroheptanoic acid. The remaining supernatant was diluted with acetonitrile (AcN) to make a final 70:30 AcN:H_2_0 solution for one carbon metabolite analysis. A modified extraction and derivatization protocol was used for polyamine analysis^34^. Briefly, frozen samples (~ 25 mg) were homogenized using a Mixer Mill 300 tissue homogenizer with a metal bead in 600 µl of ice cold 0.4 M PCA spiked with putrescine-d8, spermidine-d6 and spermine-d20 internal standards. The mixture was centrifuged at 13,000 g for 10 min at 4 °C. Supernatant (15 µl) was derivatized by adding 6% benzoyl chloride in AcN and vortexed every 15 min for 1 h at room temperature. Potassium hydroxide (2 M, KOH) was added to the tube along with 3.2 M formic acid for analysis. For creatine analysis, frozen liver samples (~ 50 mg) were homogenized using a Mixer Mill 300 tissue homogenizer with 1,000 µl of 70:30 methanol:water spiked with creatine-d5, creatinine-d3, and guanidinoacetate-d2 and a metal bead in a 2 ml polypropylene tube and centrifuged for 10 min at 13,000×*g*. The supernatant (100 µl) was dried down with nitrogen, reconstituted in 50 µl water, and added to 950 µl AcN. For extraction in serum or urine, 2 µl was added to 998 µl 95:5 AcN:water spiked with internal standards, centrifuged for 10 min at 13,000×*g*, and the supernatant was used for further analysis.

### Liquid chromatography tandem mass spectrometry

SAM and SAH were analyzed on a Waters Quattro Micro triple quadrupole mass spectrometer run in positive ionization mode with multiple reaction monitoring (MRM) attached to a Waters ultra-performance liquid chromatography (UPLC) system (Waters, Milford, MA) using a Waters Acquity HSS T3 column (1.8 µm particle size, 2.1 × 100 mm, Waters, Milford, MA) at 40 °C. Mobile phases consisted of 10 mM PFHA (solution A) and AcN (solution B). Additional OCM metabolites (betaine, cystathionine, homocysteic acid, L-methionine, and N,N-dimethylglycine) were measured on a Water TQD triple quadrupole mass spectrometer run in positive ionization mode attached to a Waters UPLC system equipped with a Waters Acquity UPLC BEH amide column (1.7 µM particle size, 2.1 × 100 mm, Waters, Milford, MA) held at 40 °C. Mobile phases consisted of 10 mM ammonium formate + 0.1% formic acid (solution A) and AcN (solution B). Creatine and derivatized polyamine extractions were analyzed on a Waters Xevo G2-XS QTof attached to a Waters UPLC system using exact mass and retention times for identification and quantification of metabolites. Extracts were separated using a Waters Acuity UPLC BEH amide column (1.7 µM particle size, 2.1 × 100 mm) held at 40 °C. Mobile phases consisted of 10 mM ammonium formate (solution A) and AcN (solution B). The QTof was run in positive ionization mode with continuum data acquisition and leucine enkephalin used as the lockspray reference compound. The MS total useful signal (MSTUS) method was used to normalize urine samples^35^. Progenesis software (Waters, Milford, MA) was used to determine total useful signal for each sample by summing metabolite peak areas common to all samples. Derivatized polyamine extracts were separated with a Waters Acquity UPLC BEH C18 column (1.7 µM particle size, 2.1 × 100 mm) held at 30 °C. The mobile phases were water containing 0.1% formic acid (solution A) and AcN (solution B). The QTof was run in positive ionization mode with continuum data acquisition and leucine enkephalin used as the lockspray reference compound. Liver extracts were normalized to total protein of each sample.

### Protein quantification and capillary electrophoresis protein analysis

Protein quantification and electrophoresis protein analysis were performed as previously published with slight modifications^33^. Briefly, Dried protein pellets from metabolic extractions were resuspended in 0.1 M KOH and quantified using a standard curve made with bovine serum albumin and the bicinchoninic acid (BCA) assay (Sigma-Aldrich). For OCM protein analysis, liver samples were homogenized in RIPA buffer supplemented with a protease inhibitor cocktail (Sigma-Aldrich) using a Polytron PT2100 homogenizer (Kinematica, Lucerne, Switzerland) and homogenized on ice. Samples were centrifuged and concentration measured using the BCA assay. The WES capillary electrophoresis system (ProteinSimple, San Jose, CA) was used with the following antibodies and dilutions from Abclonal (Cambridge, MA): AHCY (#A5300; 1:130), BHMT (#A6357; 1:300), CBS (#A1427; 1:100), GNMT (#A6608; 1:130), and MAT1A (#A6650; 1:200). Primary antibodies were detected using a polyclonal anti-rabbit secondary antibody conjugated to horseradish peroxidase. Chemiluminescence signal raw data was analyzed with the Compass Software (ProteinSimple, San Jose, CA). Target protein levels were normalized to total protein levels.

### Gene expression analysis

Hepatic RNA-seq data sets were previously published^22^. Genes were considered differentially expressed when |fold-change|≥ 1.5 and posterior probability values (P1(t)) ≥ 0.8 as determined by an empirical Bayes approach^36,37^. For figures, relative transcript counts represents the maximum raw number of aligned reads to each transcript across all treatments indicating the potential level of hepatic expression, where low level of expression ≤ 500 reads, and higher level of expression ≥ 10,000 reads. Sequencing data for the 72 h time course and 28 day dose response study are available at the Gene Expression Omnibus (GEO; GSE109863 and GSE87519, respectively).

Hepatic gene expression in the 8-day study and renal gene expression in the 28 day study were assessed using quantitative real-time polymerase chain reaction (qRT-PCR). Total RNA was reverse transcribed by SuperScript II (Invitrogen) using oligo dT primer according to the manufacturer’s protocol. PCR amplification was conducted on a Bio-Rad CFX Connect Real-Time PCR Detection System. Gene expression relative to vehicle control was calculated using the 2^−ΔΔCT^ method. Liver samples were normalized to the housekeeping genes *ActB*, *Hprt*, and *Gapdh*. Kidney samples were normalized to *Gapdh*. Primer sequences are provided in Supplementary Table S1. BMD Express 2.0^38^ was used for benchmark dose response modeling to calculate benchmark dose lower confidence limits (BMDL) using parameters defined by Yang et al.^39^. If the best fit model was sigmoidal, the ED_50_ value was calculated from sigmodal parameters used for the model fit.

### ChIP and putative DRE identification

The AhR ChIP-seq data and computationally identified putative dioxin response elements (pDREs) were previously published^40^. Significant AhR ChIP-seq binding used a false discovery rate (FDR) ≤ 0.05. pDREs were considered functional with a matrix similarity score (MSS) ≥ 0.856. ChIP-seq data is available in the Gene Expression Omnibus (GSE97634).

## Results

### TCDD elicited dose-dependent effects on OCM

AhR activation following acute or repeated treatment with TCDD elicits NAFLD pathologies in mice that include dose-dependent hepatic lipid accumulation, immune cell infiltration, and periportal fibrosis with bile duct proliferation occurring only in males^22,40–45^. Dysregulation of OCM, most notably SAM and SAH levels, is reported in human NAFLD and rodent models^2,9^. Gene expression, protein levels, and metabolite levels were integrated to further investigate the time and dose-dependent effects of TCDD on OCM including the SAM-dependent creatine and polyamine biosynthesis pathways.

**Figure 1:**
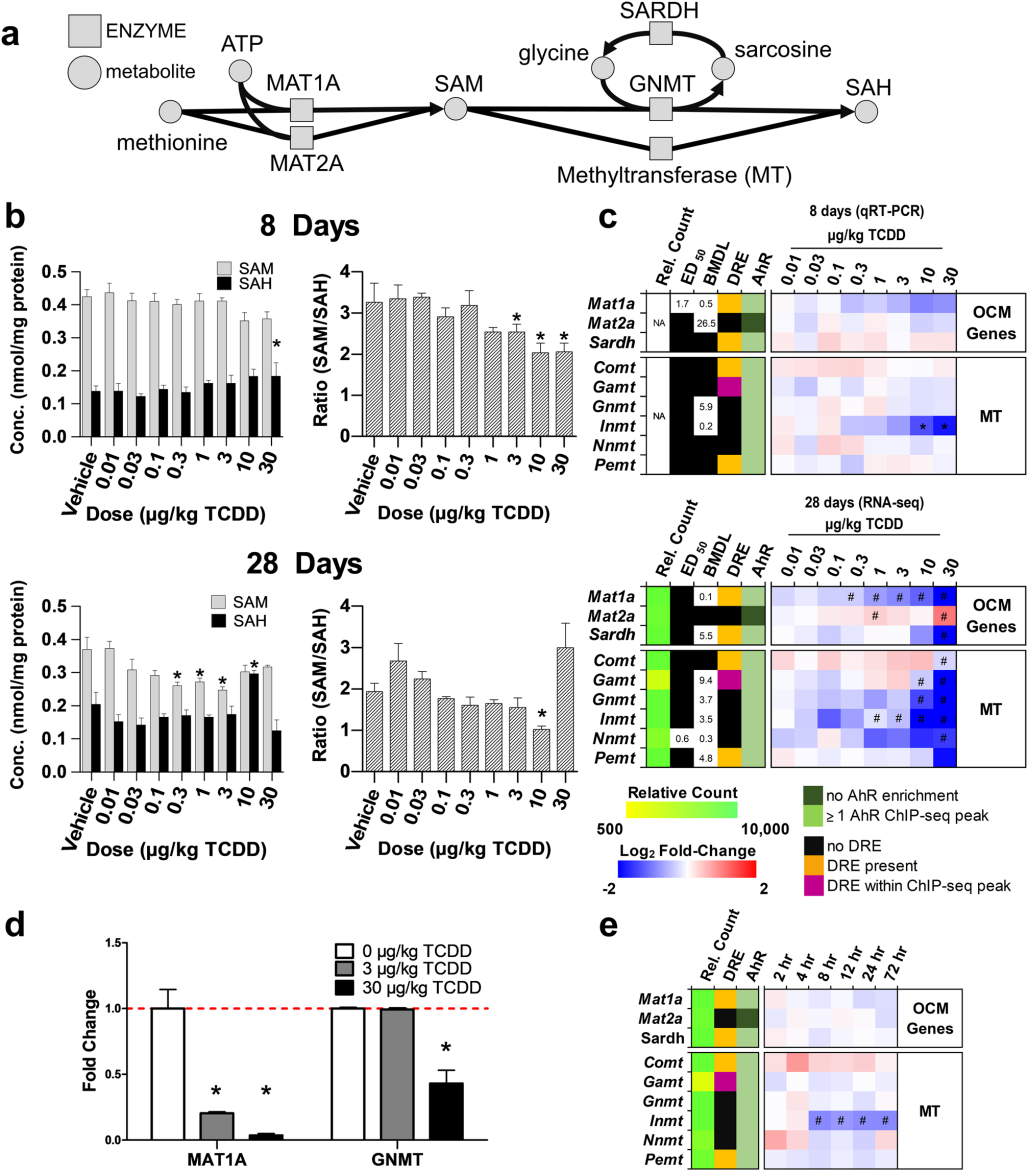
TCDD-elicited repression of SAM biosynthesis and methyltransferase gene expression. (**a**) Schematic pathway depicting enzymes (open rectangle) and metabolites (open circle) associated with SAM biosynthesis and utilization by methyltransferases (MT). (**b**) Hepatic levels of SAM and SAH were determined by LC–MS/MS (mean ± s.e.m., n = 5–6) at 8 and 28 days of repeated TCDD exposure and (**c**) hepatic gene expression of genes involved in the biosynthesis, regulation, and utilization of SAM and SAH were assessed at 8 and 28 days by RT-qPCR or RNA-seq, respectively (n = 8). (**d**) Fold change for hepatic MAT1A and GNMT protein levels after 28 days measured by the WES capillary electrophoresis system (mean ± s.e.m., n = 4). (**e**) Hepatic gene expression associated with SAM metabolism was determined by RNA-seq for a time-course after a bolus dose of 30 µg/kg TCDD (n = 5). For the heatmaps, the median effective dose (ED_50_) and benchmark dose lower limit (BMDL) and relative transcript count (rel. count, ) are denoted. The red/blue color scale represents the log_2_(fold change) for differential gene expression. Orange represents the presence of putative dioxin response elements (pDREs). AhR enrichment peaks (FDR ≤ 0.05) are denoted by light green. pDREs found within AHR ChIP-seq enrichment peaks are denoted by garnet. Asterisks (*) denote *p* < 0.05 determined by one-way ANOVA with a Dunnett’s post-hoc test. Pound signs (#) denote posterior probabilities P1(t) ≥ 0.80 compared to vehicle. Official gene name and symbol, and metabolite abbreviations: *Comt* catechol-*O*-methyltransferase, *Gamt* guanidinoacetate *N*-methyltransferase, *Gnmt* glycine *N*-methyltransferase, *Inmt* indolethylamine *N*-transferase, *Mat1a, Mat2a* S-adenosylmethionine synthase isoform 1a or 2a, *Nnmt* nicotinamide *N*-methyltransferase, *Pemt* phosphatidylethanolamine *N*-methyltransferase, *Sardh* sarcosine dehydrogenase, *SAM* S-adenosylmethionine, *SAH* S-adenosylhomocysteine.

To assess the effects of TCDD on SAM biosynthesis and metabolism (Fig. 1a), gene expression, protein levels, and metabolite levels were analyzed in liver samples after mice were orally gavaged every 4 days for 8 or 28 days. At 8 days, TCDD elicited a dose-dependent decrease in the SAM/SAH ratio (Fig. 1b). By 28 days, the SAM/SAH ratio exhibited a non-monotonic dose–response, with a decreasing trend between 0.3 and 10 µg/kg TCDD (Fig. 1c). The effects on SAM and the SAM/SAH ratio are consistent with changes in OCM gene expression and protein levels. At 8 days, TCDD dose-dependently repressed *Mat1a* (BMDL 0.5 µg/kg; Fig. 1c). At 28 days, TCDD repressed *Mat1a* 4.5-fold (BMDL 0.1 µg/kg). However, at 30 µg/kg TCDD, *Mat2a* was induced 2.1-fold while repressing highly expressed *Gnmt* (12.1-fold), a known regulator of SAM levels, and *Sardh* (19.6-fold), which catalyzes the oxidative demethylation of sarcosine back to glycine (Fig. 1c). The dose-dependent decreases in MAT1A and GNMT protein levels were in agreement with respective gene repression (Fig. 1d). In addition, other highly expressed SAM-dependent methyltransferases including guanidinoacetate N-methyltransferase (*Gamt*), indolethylamine N-methyltransferase (*Inmt)*, nicotinamide N-methyltransferase (*Nnmt*), and phosphatidylethanolamine N-methyltransferase (*Pemt*) were repressed 2.0-, 636.4-, 2.9- and 3.7-fold, respectively at 28 days (Fig. 1c). The non-monotonic dose–response for the SAM/SAH ratio at 28 days likely involves dysregulation of *Mat1a* and *Mat2a* expression, as well as the repression of *Gnmt*, in addition to the repression of SAM-dependent *Gamt*, *Inmt*, *Nnmt,* and *Pemt* methylation reactions (Fig. 1b,c). Despite pDRE-independent AhR enrichment at 2 h for most of these genes, there was negligible gene repression within the first 72 h following treatment with 30 µg/kg TCDD (Fig. 1e). Moreover, repression of the above genes was greater at 28 days compared to 8 days.

**Figure 2:**
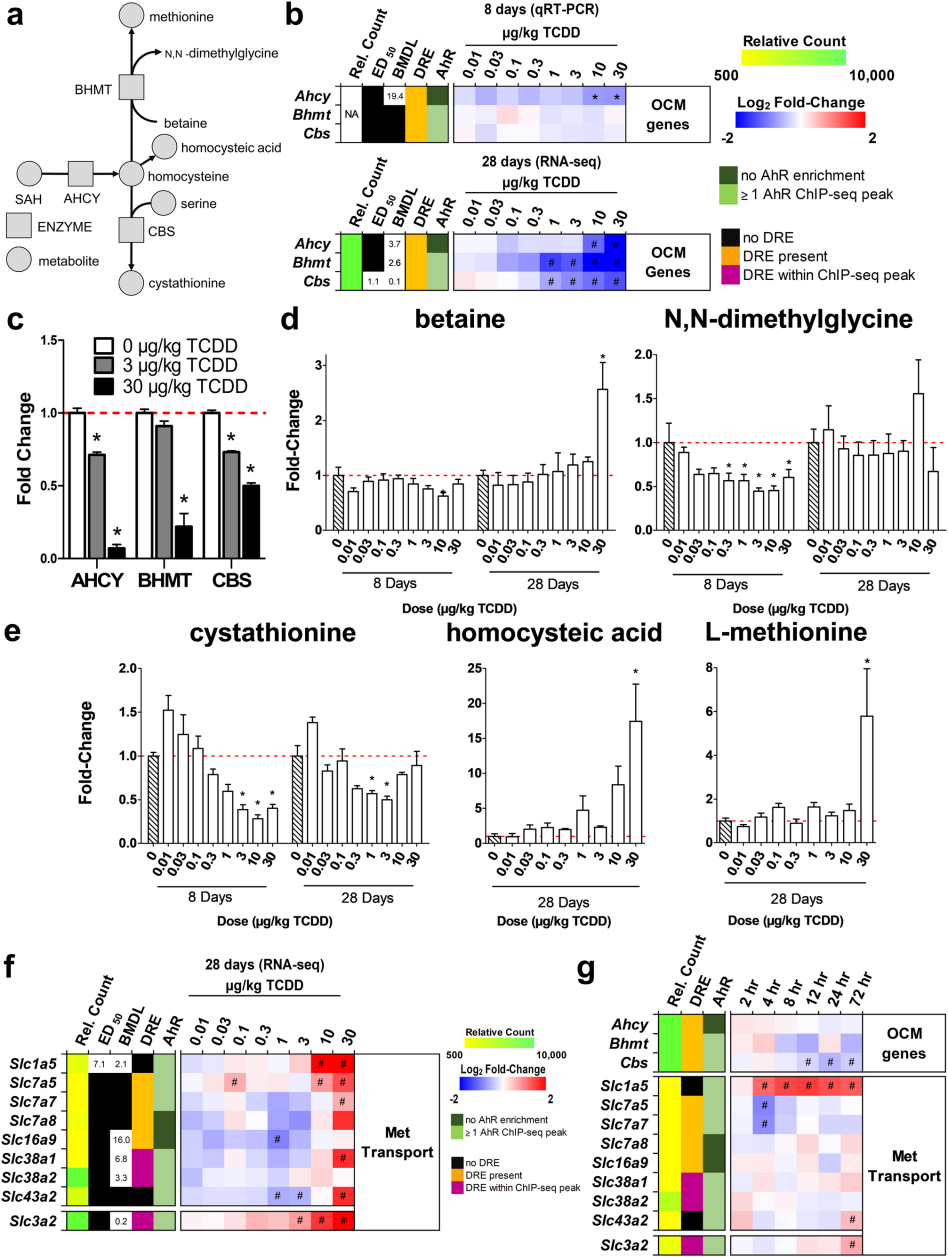
TCDD elicited effects on the hepatic metabolism of homocysteine. (**a**) Schematic of pathway depicting enzymes and metabolites associated with homocysteine metabolism. Boxes represent enzymes and circles represent metabolites. (**b**) Hepatic gene expression associated with homocysteine metabolism was measured at 8 or 28 days by qRT-PCR and RNA-seq, respectively (n = 8). (**c**) Hepatic protein levels (mean ± s.e.m.) were determined by capillary electrophoresis for AHCY, BHMT, and CBS in male mice at 28 days (n = 4). (**d**) Metabolite fold change at 8 days (mean ± s.e.m., n = 3–6) or 28 days (mean ± s.e.m., n = 4–5) were determined by LC–MS/MS for betaine, *N*,*N*-dimethylglycine and (**e**) cystathionine (8 and 28 days), or methionine and homocysteic acid (28 days only). (**f**) Hepatic gene expression of methionine transporters at 28 days (n = 8). (**g**) Hepatic gene expression associated with homocysteine metabolism was determined by RNA-seq for a time-course after a bolus dose of 30 µg/kg TCDD (n = 5). For the heatmaps, the effective dose (ED_50_), benchmark dose lower limit (BMDL), and relative transcript counts (rel. count) are denoted. The red/blue color scale represents the log_2_(fold change) for differential gene expression. Orange represents the presence of putative dioxin response elements (pDREs). AhR enrichment peaks (FDR ≤ 0.05) are denoted by light green. pDREs found within AHR ChIP-seq enrichment peaks are denoted by garnet. Asterisks (*) denote *p* < 0.05 determined by one-way ANOVA with a Dunnett’s post-hoc test. Pound signs (#) denote posterior probabilities P1(t) ≥ 0.80 compared to vehicle. Official gene name and symbol: *Ahcy* adenosylhomocysteinase, *Bhmt* betaine homocysteine S-methyltransferase, *Cbs* cystathionine beta-synthetase.

Other metabolites of the OCM and transsulfuration pathways (Fig. 2a) are important for methylation and were also affected by TCDD. Homocysteine is the product of SAH hydrolysis catalyzed by adenosylhomocysteine (AHCY) which was dose-dependently repressed by TCDD at the mRNA and protein levels in the absence of AhR enrichment (Fig. 2b). Under normal conditions, BHMT uses betaine as a donor to methylate homocysteine in the re-synthesis of methionine, producing N,N-dimethylglycine as a byproduct. At 28 days, *Bhmt* mRNA and protein levels were dose-dependently repressed by TCDD (Fig. 2b,c). Accordingly, there was an 2.6-fold increase in betaine and a non-significant 1.4-fold decrease in *N*,*N*-dimethylglycine (Fig. 2d). Alternatively, homocysteine can enter the transsulfuration pathway. However, cystathionine β-synthase (*Cbs*) mRNA and protein levels were also dose-dependently repressed with a corresponding decrease in cystathionine levels (Fig. 2b,c,e). At 28 days, cystathionine levels recovered following treatment with 10 and 30 µg/kg TCDD which may be due the allosteric activation of CBS by increasing SAM levels. Repression of BHMT in the methionine cycle and CBS in the transsulfuration pathway is consistent with the dose-dependent increase in homocysteic acid (Fig. 2e), produced as a result of the spontaneous oxidation of accumulating homocysteine^46^. Collectively, these changes would be expected to reduce hepatic methionine levels, but they were increased 5.8-fold (Fig. 2e). This may be due to increased methionine import with the induction of *Slcs 1a5*, *7a5*, *7a7*, *7a8*, *38a1*, *38a2* and *43a2*. *Slc3a2*, the heavy chain heterodimeric partner for many amino acid transporters, was also dose-dependently induced by TCDD and contained a ChIP-seq peak with a pDRE (Fig. 2f).

As observed with *Mat1a*, *Mat2a*, *Gnmt*, *Gamt*, *Inmt* and *Pemt*, the repression of *Ahcy* and *Bhmt* and *Cbs* was also negligible within the first 72 h following treatment with 30 µg/kg TCDD (Fig. 2g). *Bhmt*, *Cbs, Slc1a5, 3a1, 7a5, 7a7, 7a8, 16a9,* and *43a2* also exhibited pDRE-independent AhR enrichment at 2hrs, while the ChIP-seq peaks for *Slc38a1* and *Slc38a2* did contain a pDRE within the enriched AhR bound region.

### Effects on polyamine metabolism

**Figure 3:**
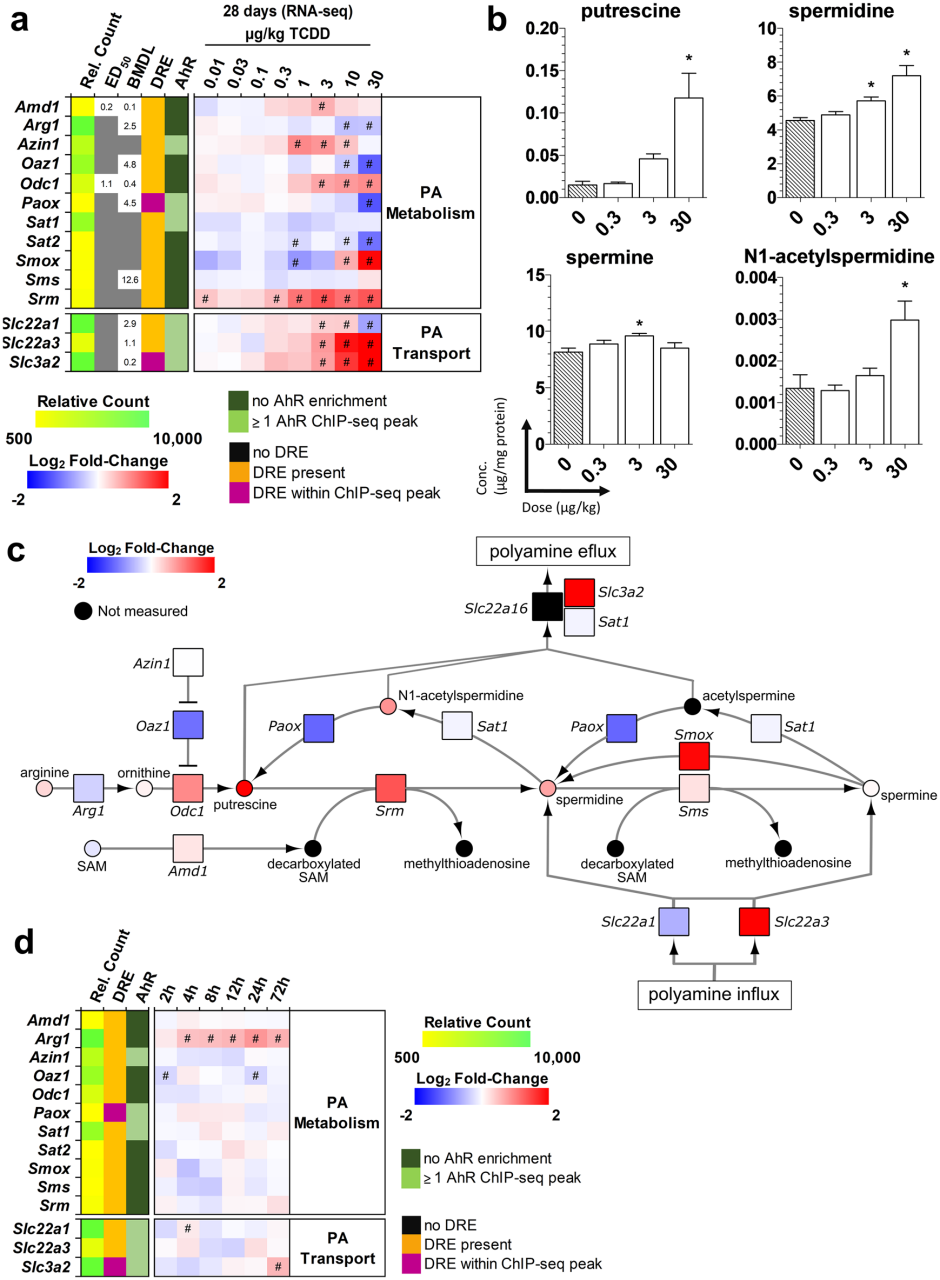
TCDD-Elicited Effects on Polyamine (PA) Biosynthesis. (**a**) Hepatic gene expression associated with PA metabolism was examined using RNA-seq at 28 days repeated TCDD exposure (n = 8). (**b**) Hepatic PA levels were determined by LC–MS/MS at 28 days repeated TCDD exposure (mean ± s.e.m., n = 5). (**c**) Hepatic gene expression associated with polyamine metabolism was determined by RNA-seq for a time-course after a bolus dose of 30 µg/kg TCDD. (**d**) Schematic pathway of hepatic polyamine biosynthesis incorporating fold changes of metabolites (open circle) and gene expression (open rectangle) in male mice orally gavaged with sesame oil vehicle or 30 µg/kg TCDD every 4 days for 28 days. Fold changes of metabolites and gene expression were determined by LC–MS/MS or RNA-seq, respectively. For the heatmaps, the effective dose (ED_50_), benchmark dose lower limit (BMDL), and relative transcript count (Rel. Count) are denoted. The red/blue color scale represents the log_2_(fold change) for differential gene expression. Orange represents the presence of putative dioxin response elements (pDREs). AhR enrichment peaks (FDR ≤ 0.05) are denoted by light green. pDREs found within AHR ChIP-seq enrichment peaks are denoted by garnet. Asterisks (*) denote *p < 0.05 determined by one-way ANOVA with a Dunnett’s post-hoc test. Pound signs (#) denote posterior probabilities P1(t) ≥ 0.80 compared to vehicle.). Official gene name and symbol, and metabolite abbreviations: *Amd1* S-adenosylmethionine decarboxylase, *Azin1* antizyme inhibitor 1, *Oaz1* ornithine decarboxylase antizyme 1, *Odc1* ornithine decarboxylase, L-ornithine (ORN), *Paox* peroxisomal N1-acetyl-spermine/spermidine oxidase, *PA* polyamine, *Sat1 & 2* spermine/spermidine acetyltransferase, *Slc3a2* 4F2 cell-surface antigen heavy chain, *Srm* spermidine synthase, *Sms* spermine synthase, *Smox* spermine oxidase, *SAM* S-adenosylmethionine, *Decarbox-SAM* decarboxylated SAM, *MTA* methylthioadenosine.

To further investigate the effects of TCDD-elicited alterations in the SAM/SAH ratio, subsequent effects on SAM-dependent pathways were examined. Polyamines are ubiquitous polycationic alkylamines that are crucial for a broad range of cellular functions including cell cycle modulation, scavenging reactive oxygen species, and the control of gene expression. S-adenosylmethionine decarboxylase (*Amd1*), which was modestly induced (1.6-fold at 3 µg/kg TCDD, Fig. 3a), catalyzes the decarboxylation of SAM to produce decarboxylated SAM. The donation of the propylamine group from decarboxylated SAM to putrescine is catalyzed by spermine synthase (*Srm*) which was induced 2.5-fold at 30 µg/kg TCDD (Fig. 3a). Surprisingly, putrescine levels, which are typically kept low within cells^47^, were increased 8.0-fold at 30 µg/kg TCDD (Fig. 3b), possibly due to the 1.9-fold induction of ornithine decarboxylase (*Odc1*), and the 2.3-fold repression of ornithine decarboxylase antizyme (*Oaz1*), which promotes ODC1 degradation (Fig. 3a)^5^. There were also 1.6- and 2.2-fold increases in spermidine and N1-acetylspermidine at 30 µg/kg TCDD, respectively (Fig. 3b). The 4.9-fold induction of the polyamine transporter, *Slc22a3*, and the 3.9-fold induction of spermine oxidase (Smox) at 30 µg/kg TCDD, which oxidizes spermine to spermidine, may also contribute to putrescine accumulation (Fig. 3a). Like methionine and homocysteine metabolism, polyamine associated gene expression exhibited only moderate changes in the first 72 h following a bolus dose of 30 µg/kg TCDD (Fig. 3c). Collectively, TCDD dysregulated polyamine biosynthesis and transport, consistent with increased putrescine, spermidine and N1-acetylspermidine levels (Fig. 3d).

### Effects on creatine metabolism

**Figure 4:**
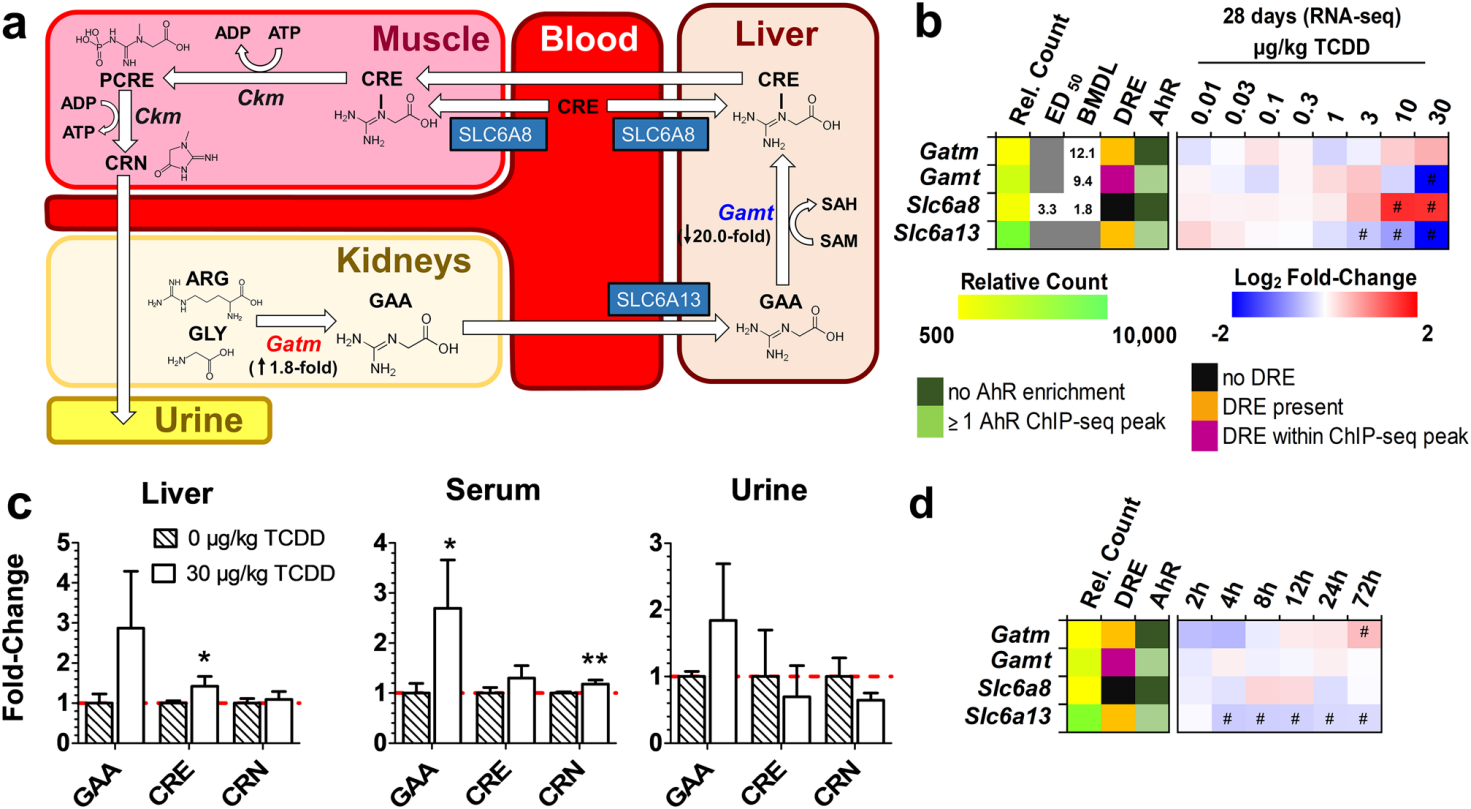
TCDD-Elicited Dose-Dependent Effects on Creatine Biosynthesis. (**a**) Schematic of systemic creatine biosynthesis and transport. For 30 µg/kg TCDD treatment groups, renal *Gatm* expression and hepatic *Gamt* fold-changes from vehicle are denoted. (**b**) Hepatic gene expression associated with creatine metabolism at 8 or 28 days repeated TCDD exposure. (**c**) Guanidinoacetate (GAA)-, creatine (CRE)-, and creatinine (CRN)-fold changes determined by LC–MS/MS in male mice orally gavaged with sesame oil vehicle or 30 µg/kg TCDD every 4 days for 28 days (mean ± s.e.m., n = 5). (**d**) Hepatic expression associated with creatine metabolism in male mice orally gavaged with a bolus dose of 30 µg/kg TCDD (n = 8). For the heatmaps, the effective dose (ED_50_), benchmark dose lower limit (BMDL), and relative transcript count (Rel. Count) are denoted. The red/blue color scale represents the log_2_(fold change) for differential gene expression. Orange represents the presence of putative dioxin response elements (pDREs) with a matrix similarity scores (MSS) ≥ 0.856. AhR enrichment peaks (FDR ≤ 0.05) denoted by light green were determined by ChIP-seq. pDREs found within AHR ChIP-seq enrichment peaks are denoted by garnet. Asterisks denote (*) *p* < 0.05 or (**) p < 0.01 determined by one-way ANOVA with a Dunnett’s post-hoc test. Pound signs (#) denote posterior probabilities P1(t) ≥ 0.80 compared to vehicle. Official gene name and symbol, and metabolite abbreviations: *Gamt* guanidinoacetate *N*-methyltransferase, *Gatm* glycine amidinotransferase, mitochondrial, *Ckm* creatine kinase M-type, *ARG* arginine, *GLY* glycine, *GAA* guanidinoacetate, *CRE* creatine, and *CRN* creatinine, *PCRE* phosphocreatine.

The consequences of OCM dysregulation on SAM-dependent creatine biosynthesis were also assessed. Creatine biosynthesis begins in the kidneys where mitochondrial glycine amidinotransferase (*Gatm* aka *Agat*) transfers a guanidino group from arginine (ARG) to glycine (GLY) to produce guanidinoacetate (GAA) (Fig. 4a). In the liver, GAA is methylated to form creatine by guanidinoacetate *N*-methyltransferase (*Gamt*) which is reported to consume 60% of hepatic SAM^48^. At 28 days, TCDD increased renal *Gatm* (1.8-fold) and decreased hepatic *Slc6a13* (6.3-fold), a guanidinoacetate transporter^49^, and hepatic *Gamt* (20.0-fold) (Fig. 4a,b). This coincided with increases in serum GAA levels (Fig. 4c). The modest 1.3-fold increase in hepatic creatine levels was accompanied by a 3.0-fold increase in the creatine importer *Slc6a8* (ED_50_: 3.4 µg/kg TCDD, 3.0-fold), while urinary creatinine decreased 1.5-fold (trending). Despite DRE-dependent AhR enrichment in *Gamt*, no changes in gene expression were observed up to 72 h following treatment (Fig. 4d). These results suggest TCDD-elicited repression of *Gamt* caused systemic increases in GAA levels at 28 days.

## Discussion

Alterations in OCM, including human polymorphisms, correlate with NAFLD progression and severity^50–52^. Previous work has shown that persistent AhR activation by TCDD induces a NAFLD-like phenotype that includes hepatic fat accumulation, inflammation, and mild fibrosis in mice^22,41,45,53^. However, the effects of TCDD on OCM within the context of this NAFLD model have not been comprehensively investigated. Herein, we show TCDD dose-dependently alters OCM at the transcript, protein, and metabolite levels. The SAM/SAH ratio, an indicator of methylation potential, and creatine and polyamine biosynthesis pathways were altered.

In this study, TCDD is used as a surrogate for the cumulative burden of all AhR ligands. Mice were orally gavaged with 0.01–30 µg/kg TCDD starting at post-natal day 28 with TCDD every 4 days for 8 and 28 days to approach steady state levels due to the 8–12 day half-life of TCDD in mice^24,54^. Using this dosing regimen, oral gavage of 30 µg/kg TCDD resulted in mouse hepatic tissue levels comparable to serum levels reported in Viktor Yushchenko following intentional poisoning, while 0.01 µg/kg TCDD increased hepatic levels compared to background levels in control mouse liver, and were comparable to the level of dioxin-like compounds in the serum of US, German, Spanish and United Kingdom populations^23,55–60^. Similar doses and/or treatment regimens have been used in previous studies from this lab as well as other groups^33,44,61–63^. Although there was an increase in serum ALT levels following oral gavage with 30 µg/kg TCDD every 4 days for 28 days for a total of 7 treatments, there was no evidence of overt toxicity, no body weight loss > 15%, no significant change in food consumption, and no histopathological evidence of necrosis or apoptosis^23,33,64^. Consequently, the dose-dependent effects of TCDD on OCM, the SAM/SAH ratio and the biosynthesis of polyamines and creatine cannot be attributed to overt toxicity.

**Figure 5:**
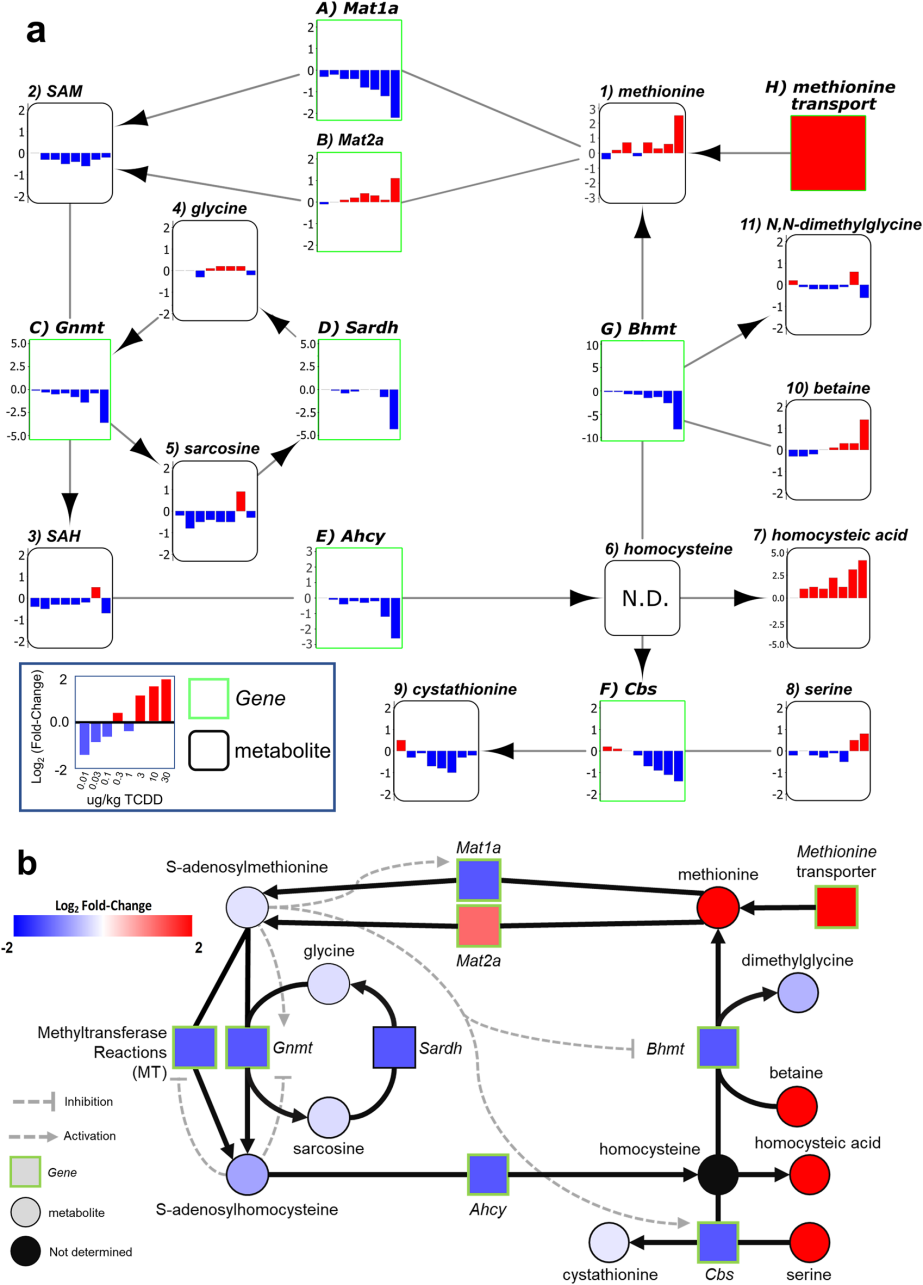
Summary of effects of TCDD on OCM gene expression and metabolites. (**a**) OCM pathway schematic depicting dose response effects of TCDD on gene expression (green rectangles labelled A–H) and metabolite levels (black rounded rectangles labelled 1–11) in male C57BL/6 mice at 28 days repeated TCDD exposure. Gene expression and metabolite levels were determined by RNA-seq or LC–MS/MS, respectively. Homocysteine levels were not determined (N.D.). The Log_2_(fold change) range is denoted on left y-axis of each box and the bar colors represents red as induced and blue as repressed. (**b**) OCM pathway schematic depicting changes in gene expression and metabolite levels in male C57BL/6 mice orally gavaged with sesame oil vehicle or 30 µg/kg TCDD every 4 days for 28 days. Inhibition or activation of enzymes in pathway are indicated by grey dashed lines. Changes in genes expression and metabolite levels were determined by RNA-seq or LC–MS/MS, respectively. Green boxes represent genes and black circles represent metabolites. The Log_2_(fold change) represents red as induced and blue as repressed. Official gene name and symbol: (a) *Mat1a* S-adenosylmethionine synthase isoform 1a, (b) *Mat2a* S-adenosylmethionine synthase isoform 2a, (c) *Gnmt* glycine *N*-methyltransferase, (d) *Sardh* sarcosine dehydrogenase, (e) *Ahcy* adenosylhomocysteinase, (f) *Cbs* cystathionine beta-synthetase, (g) *Bhmt* betaine homocysteine methyltransferase, (h) *Slc1a5, 7a5, 7a7, 7a8, 16a9, 38a1, 38a2, and 43a2* solute carrier family.

*Mat1a* is highly expressed in the adult liver making it the major site of SAM biosynthesis. As the primary methyl group donor for methyltransferase reactions, hepatic SAM and SAH levels are maintained in a narrow range with increases or decreases to the SAM/SAH ratio outside this window potentially affecting numerous cell functions^2,65^. Our studies show TCDD dose-dependently repressed *Mat1a* mRNA and protein levels (Fig. 1c,d), as previously reported with other AhR ligands^66,67^. TCDD also altered the SAM/SAH ratio with trends suggesting SAM levels decreased while SAH levels increased at higher doses at 8 days (Fig. 1b). A similar trend was observed at 28 days, except for a reproducible increase in the SAM/SAH ratio at 30 µg/kg TCDD (Fig. 1b) that coincided with the induction of *Mat2a* and the repression of several highly expressed methyl transferases including GNMT (Fig. 5a). GNMT is the most abundant hepatic methyltransferase that acts as a sink by transferring methyl groups from SAM to glycine to reduce SAM levels in order to regulate methionine consumption and SAM levels^7^. Interestingly, repression of *Mat1a* with the induction of *Mat2a*, which is usually only expressed during liver development, is characteristic of NAFLD and aggressive hepatocellular carcinoma progression^68–72^.

SAH is a potent competitive methyltransferase inhibitor^73^. It is readily hydrolyzed by AHCY to adenosine and homocysteine (not measured in our analysis) to allow OCM to proceed. However, AHCY mRNA and protein levels were dose-dependently repressed by TCDD (Fig. 2b,c). Homocysteine can be re-methylated back to methionine by BHMT or MTR which catalyzes the transfer of a methyl group from betaine or 5-methyltetrahydrofolate, respectively. In mice, BHMT is highly expressed in the liver and is the primary methionine biosynthesis pathway^74^. TCDD dose-dependently repressed BHMT coincident with an increase in betaine and decrease in *N*,*N*-dimethylglycine (Fig. 2c,d). An alternative pathway to re-methylation, homocysteine can be converted to cystathionine by CBS, which would undergo further metabolism by cystathionine gamma-lyase to produce cysteine and support glutathione biosynthesis. However, TCDD dose-dependently repressed *Cbs* mRNA and protein levels, and reduced cystathionine levels (Fig. 2b,c,e). CBS activity may also be further allosterically repressed by TCDD induced oxidative stress^75,76^. The partial recovery of cystathionine levels at higher TCDD doses is consistent with *Mat2a* induction with increased SAM levels allosterically activating CBS^6^. Consequently, homocysteine is not consumed in the transsulfuration pathway or by re-methylation to methionine, and undergoes oxidation as indicated by the increase in homocysteic acid levels, the spontaneous oxidation product of homocysteine (Fig. 2e). Despite BHMT transcriptional repression and inhibition by SAM, hepatic methionine levels increased due to the induction of transporters, and the repression of *Mat1a* and major methyltransferases that consume SAM (Fig. 5).

Given TCDD affected OCM, we next examined the potential consequences of an altered SAM/SAH ratio on polyamine and creatine biosynthesis. Polyamines are low molecular weight aliphatic polycations present in all living cells. De novo synthesis, interconversion, degradation and transport ensure levels of putrescine, spermidine and spermine are maintained in a narrow range since low levels inhibit cell proliferation and high levels or catabolic byproducts are toxic^5,47^. Approximately 5% of hepatic SAM is used to produce polyamines^48,77^. Consistent with previous reports that TCDD increases ODC activity, our results showed TCDD increased *Odc* expression and disrupted polyamine biosynthesis^78–80^, in contrast to short term studies that reported TCDD decreased polyamine levels^81–83^. Adaptive compensatory responses such as increased *Smox* expression, catalyzing the interconversion of spermine to spermidine, and the differential expression of transporters, may partially explain the different effects of TCDD on polyamine levels we observed after 28 days of treatment (Fig. 3). The increase in putrescine by TCDD is comparable to levels induced by 12-O-tetradecanoylpphorbol-13-acetate (TPA), an inducer of ODC activity^84–86^. Putrescine levels are normally low when demand for polyamines are low due to multiple levels of ODC activity regulation and the allosteric activation of SAM decarboxylase (AMD1)^5^. Yet, putrescine levels increased despite the TCDD-elicited dose-dependent induction of *Odc* and spermidine synthase (*Srm*) suggesting decarboxylated SAM may be limiting (Fig. 3a,b). Paradoxically, there was an increase in spermidine and N1-acetylspermidine levels with negligible effects on spermine levels possibly due to interconversion and/or transport to maintain cellular polyamine homeostasis.

Phosphocreatine is an important phosphate donor that can quickly regenerate ATP via substrate level phosphorylation reactions. As much as 70% of hepatic SAM is consumed in creatine biosynthesis, although hepatic levels are low with > 90% of creatine stored in muscle^87^. TCDD increased renal *Gatm* expression while repressing hepatic *Gamt* with modest effects on the levels of creatine and creatinine despite an increase in serum GAA (Fig. 4) comparable to levels reported in GAMT^−/−^ mice and humans deficient in GAMT activity^88,89^. TCDD also decreased creatinine levels in short-term in vitro studies^82,90^. Similar to polyamines, adaptive responses following prolonged TCDD exposure may account for the modest changes in hepatic creatine and creatinine levels despite the repression of *Gamt.* For instance, induction of the creatine importer, *Slc6a8,* in the liver was only observed after 28 days of treatment (Fig. 4b). Collectively, the SAM-dependent biosynthesis of both creatine and polyamine demonstrated differential gene expression and metabolite levels. Despite disruption of OCM by TCDD, the effects on polyamine and creatine biosynthesis cannot be adequately explained due to alterations on the SAM/SAH ratio alone.

Many genes associated with OCM and the transsulfuration pathway exhibited BMDLs in the sub to low µg/kg range only after 8 and 28 days of treatment. In addition, OCM and transsulfuration pathway disruption was time-dependent with the greatest effects after 28 days, more modest changes at 8 days and modest effects following a single bolus dose. The canonical mechanism of action of TCDD and related compounds involves binding to the cytoplasmic AhR, translocation to the nucleus, and heterodimerization with ARNT. The ligand-bound AhR/ARNT complex then binds to DREs within the promoter region of target genes, leading to recruitment of transcriptional co-regulators and differential gene expression^91^. Numerous studies have also reported differential gene expression following AhR binding within DNA regions lacking a DRE^92–94^. Despite ChIP-seq evidence of AhR enrichment at 2 h, only modest changes in OCM gene expression were observed in the first 72 h after treatment. In contrast, AhR targets such as *Cyp1a1, Cyp1a2* and *Tiparp* were induced within 2 h^95^. Moreover, many differentially expressed OCM genes exhibited AhR genomic enrichment in the absence of a pDRE. Collectively, these results suggest that (i) although AhR activation is required, it in itself is not sufficient and likely requires unknown additional responses, (ii) TCDD-elicited OCM disruption involves DRE-dependent and –independent changes in gene expression, and (iii) the effects of TCCD are not immediate and require persistent AhR activation.

Given that OCM and transsulfuration pathway enzyme activity is subject to allosteric activation and competitive inhibition by intermediate metabolites (Fig. 5b), and are regulated by post-translational modification, more integrative approaches such as tracer studies are required to identify the key steps affected by TCDD that alter the flux of ^13^C-labelled intermediates through OCM and its associated pathways. Moreover, an examination of other SAM-dependent reactions would expand our understanding of additional OCM mechanisms disrupted by TCDD such as the methylation of histones, DNA, and RNA associated with epigenetic regulation, and the biosynthesis of phosphatidylcholine via the PEMT and Kennedy pathways. Phosphatidylcholine is not only critical for membrane integrity, but also the secretion of very low- density lipoprotein (VLDL)^2,65^. Interestingly, the inhibition of VLDL secretion by TCDD contributes to steatosis in mice^41,53^. Additional studies are required to determine the relevance of these effects in humans due to the species-specific effects of TCDD and related compounds.

## Supplementary information

Supplementary file1.
