# Supplementary material for: 2,3,7,8-Tetrachlorodibenzo-p-dioxin (TCDD) dysregulates hepatic one carbon metabolism during the progression of steatosis to steatohepatitis with fibrosis in mice
Source: Sci Rep. 2020 Sep 9;10:14831. doi: 10.1038/s41598-020-71795-0 (PMC7481292; doi:10.1038/s41598-020-71795-0)
Supplement: Supplementary file 1 — Supplementary file1. [file 41598_2020_71795_MOESM1_ESM.pdf]

2,3,7,8-Tetrachlorodibenzo-*p*-dioxin (TCDD) Dysregulates Hepatic One Carbon Metabolism During the Progression of Steatosis to Steatohepatitis with Fibrosis in Mice

Russell R. Fling[1,3], Claire M. Doskey[2,3], Kelly A. Fader[2,3], Rance Nault[2,3], Tim R. Zacharewski[2,3]

[1]Microbiology and Molecular Genetics, Michigan State University, East Lansing, MI 48824

[2]Biochemistry and Molecular Biology, Michigan State University, East Lansing, MI 48824

[3]Institute for Integrative Toxicology, Michigan State University, East Lansing, MI 48824

Russell Fling

E-mail: flingrus@msu.edu

Claire Doskey

E-mail: CDoskey@dow.com

Kelly Fader

E-mail: kelly.a.fader@gmail.com

Rance Nault

E-mail: flingrus@msu.edu

Timothy R. Zacharewski, Ph. D.*

Telephone: (517) 355-1607

E-mail: tzachare@msu.edu

Table S1. Forward and reverse primer sequences ((5' -> 3') used for qRT-PCR analysis.

Gene Symbol	Ref Seq Accession	Forward Primer	Reverse Primer	Amplicon Length (bp)	Expected Amplification Efficiency
Ahcy	NM_016661.3	ACCAGATGTCCCATCGCTTT	GCTCAGTGGGTAAGACCGTT	166	< 1.50
Bhmt	NM_016668.3	GGCTAGGGAACTCTAGCTGC	GGTGCCATCTTTCCGGTGTA	129	1.96
Cbs	NM_144855.3	ATCCAATCACGAGACCAGGC	GCAGTGACAACCCCAAACAC	104	>2.00
Comt	NM_001111062.1	TTCTGGCCCATAAATGCTGTTG	TGCACGAACTCAAACCAACC	120	<1.50
Gamt	NM_010255.4	TTATTAAGAATCATGCCTTCCGCT	AGGCACCTGCGTCTCCTC	134	<1.50
Gapdh	NM_008084.3	GTGGACCTCATGGCCTACAT	TGTGAGGGAGATGCTCAGTG	127	1.87
Gatm	NM_025961.5	TGAAGACAAGGCCACCCATC	TATTGGCCTTCACCTCCACTG	140	1.61
Gnmt	NM_010321.1	GAAGAGGGCTTCAGCGTGAT	GCACATCTTTGTCCAGCGTC	148	>2.00
Inmt	NM_009349.3	CCTTCTCTACAGGAGGTGTAGG	GTTCTGCGGGGTGTAGTCAG	128	>2.00
Mat1a	NM_133653.3	AGGAGCTGAAGGAAGTCCGA	GGGCAAAGAGGGAGATAGCG	196	1.98
Mat2a	NM_145569.5	CTGGGGTCATTGTCAGGGAT	CCTACGCCAACAAGTCTGGG	157	1.74
Nnmt	NM_001311062.1	AATCAAGCAGGAACCTGGGC	CAATGGTGTAACCGGCCTCT	165	1.68
Pemt	NM_001290011.1	AATATCGACTTCAGGCAGGCT	GAATAGCAGGCTAGGTGGGG	197	< 1.50
Sardh	NM_138665.2	GGCACTGTTTGGACTTGCAG	AGGGCTCCTGTACCTTAGTCT	125	1.60
